# Transformation of adrenal medullary chromaffin cells increases asthmatic susceptibility in pups from allergen-sensitized rats

**DOI:** 10.1186/1465-9921-13-99

**Published:** 2012-11-08

**Authors:** Jun-Tao Feng, Xiu-Ming Wu, Xiao-Zhao Li, Ye-Qiang Zou, Ling Qin, Cheng-Ping Hu

**Affiliations:** 1 Department of Respiratory Medicine, Xiangya Hospital, Central South University, Changsha, China; 2 Department of Respiratory Medicine, First People’s Hospital of Changde, Changde, China

**Keywords:** Maternal asthma, Offspring, Susceptibility, Adrenal medulla chromaffin cell, Transformation

## Abstract

**Background:**

Studies have shown that epinephrine release is impaired in patients with asthma. The pregnancy of female rats (dams) with asthma promotes in their pups the differentiation of adrenal medulla chromaffin cells (AMCCs) into sympathetic neurons, mediated by nerve growth factor, which leads to a reduction in epinephrine secretion. However, the relatedness between the alteration of AMCCs and increased asthma susceptibility in such offspring has not been established.

**Methods:**

In this study, we observed the effects of allergization via ovalbumin on rat pups born of asthmatic dams.

**Results:**

Compared to the offspring of untreated controls, bronchial hyperreactivity and airway inflammation were more severe in the pups from sensitized (asthmatic) dams. In pups exposed to nerve growth factor (NGF) in utero these effects were aggravated further, but the effects were blocked in pups whose dams had been treated with anti-NGF. Furthermore, alterations in AMCC phenotype corresponded to the degree of bronchial hyperreactivity and lung lesions of the different treatment groups. Such AMCC alterations included degranulation of chromaffin granules, reduction of epinephrine and phenylethanolamine-n-methyl transferase, and elevation of NGF and peripherin levels.

**Conclusions:**

Our results present evidence that asthma during the pregnancy of rat dams promotes asthma susceptibility in their offspring, and that the transformation of AMCCs to neurons induced by NGF plays an important role in this process.

## Background

It is well known that maternal asthma is a risk factor for asthma in offspring [1,2]. However, details of a causal mechanism remain unknown. Results from epigenetic studies have suggested that certain environmental exposures in utero are closely related to an asthmatic [3,4]. Our recent data suggest that maternal asthma during pregnancy promotes adrenal medulla chromaffin cells (AMCCs) to differentiate into sympathetic neurons in rat pups. This in turn inhibits the maturity of the adrenal medulla, resulting in a decline in epinephrine (EPI) [5].

EPI is mainly secreted from AMCCs. AMCCs and sympathetic neurons originate from the same area in the neural crest, and it is well established that AMCCs retain the ability to transform from an endocrine to a neuronal phenotype in the presence of nerve growth factor (NGF) [6-8]. Sympathetic neurons synthesize and secrete norepinephrine and dopamine but not EPI. As a result, the secretion of EPI is impaired in transformed AMCCs, which leads to a decrease in EPI levels in the circulation.

Asthmatic attack is strongly related to dysfunctions in excitation and restraint of cholinergic nerves, adrenergic nerves, and their receptors. Human airway smooth muscles lack adrenergic nerves; the relaxation of airways is mainly regulated by EPI in circulation, which binds to the adrenergic receptors in airway smooth muscles. Studies have indicated that EPI levels were decreased in asthma patients [9,10]. On the other hand, NGF levels were found increased in asthma patients. Moreover, NGF expression appeared to be correlated with the degree of bronchial hyper reactivity [11]. Consistent with these results, our own previous studies indicated that the decline in EPI in asthma resulted from the transition of AMCCs to nerve cells, contributed to by elevated NGF [12,13].

Currently, it is not known whether the alterations in AMCCs and asthma susceptibility of offspring from an asthmatic mother are related. In this study, the effects of ovalbumin (OVA) allergization on the offspring of female rats who themselves had OVA-induced asthma were investigated. We showed that maternal asthma during pregnancy promoted susceptibility to asthma in rat pups, and that the transformation of AMCCs to neurons induced by NGF exposure in utero played an important role in this process.

## Methods

### Experimental animals and preparation

All rats used in this study were from the Experimental Animal Center of Central South University (Changsha, China). This study was carried out in strict accordance with the recommendations of the Guide for the Care and Use of Laboratory Animals published by the National Institutes of Health. The Ethics Committee of Asthma Research Institute, Hunan Province, China approved this study’s protocol.

Thirty-two pregnant Sprague–Dawley rats were randomly divided into four groups (*n* = 8 per group): control pregnant rats (CP), asthmatic pregnant rats (AP), NGF-treated pregnant rats (NP), and anti-NGF-treated pregnant rats (ANP). The rats were treated as in our previous study[5]: on gestational days 0 and 7, all groups except the control rats were sensitized with an intraperitoneal (IP) injection of 100 mg of chicken OVA (Sigma, USA), 200 mg of aluminum hydroxide (Sigma, USA), and 6 × 10^9^ heat-killed *Bordetella pertussis* (Wuhan Institute of Biological Products, China) in 1 mL of sterile saline. On the same days, the control rats were sham-sensitized without OVA. Subsequently, the OVA-sensitized rats were exposed to 30 min of 1% aerosol OVA (w/v) every day from days 14 to 21, while the control rats received filtered air only.

Thirty minutes prior to the inhalation treatments, rats in the NGF-treated group were IP-injected with NGF-7S (8 ng/kg, Sigma, USA, N0513) in phosphate buffered saline (PBS; 4 mL/kg). Those rats in the anti-NGF-treated group were IP-injected with anti-NGF (1:2000 dilution, Millipore, USA, 04–1119) in the same concentration of vehicle [13,14]. The control and asthmatic rats received the vehicle only.

**Figure 1:**
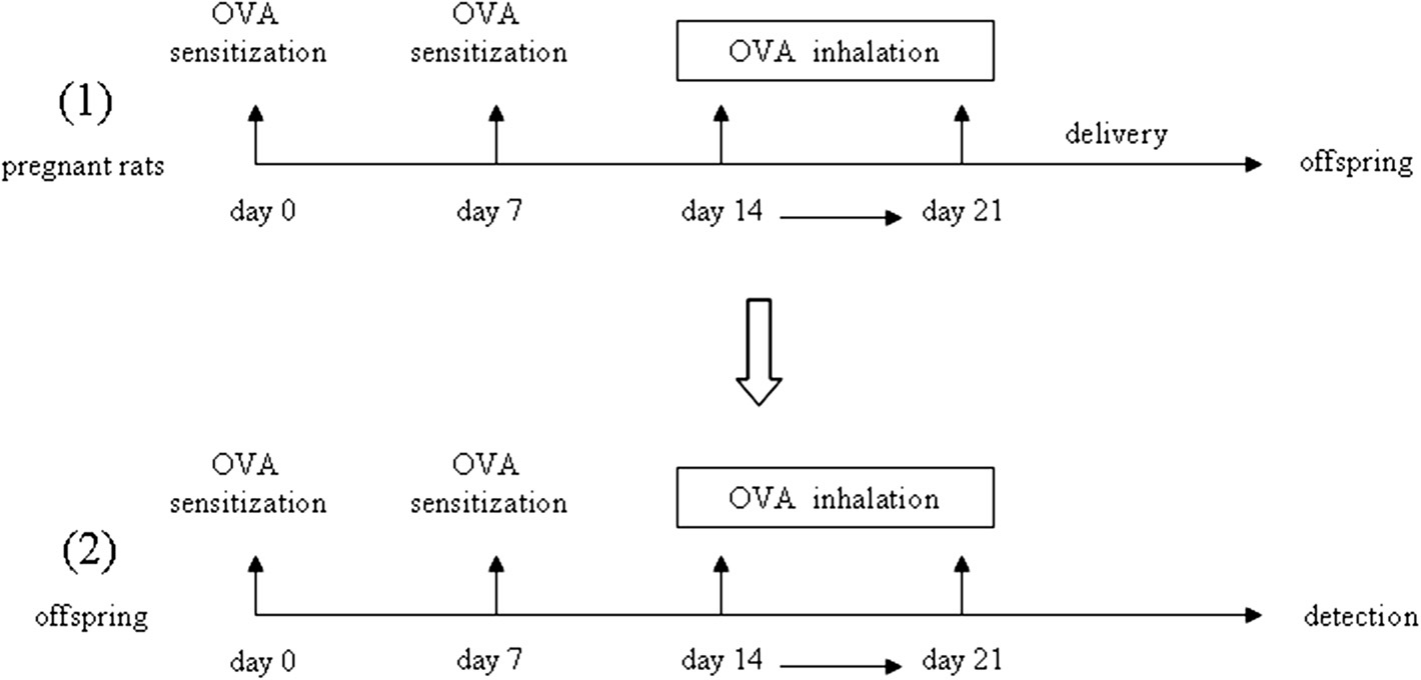
**Schematic of asthmatic models in offspring rats from asthmatic dams.** On gestational days 0 and 7, the rats were sensitized with an intraperitoneal (IP) injection of OVA. Subsequently, the OVA-sensitized rats were exposed to 30 min of 1% aerosol OVA every day from days 14 to 21. After delivery, the offspring rats were conventionally reared. Then, the offspring (6 to 8 weeks of age) were sensitized on days 0 and 7 with an IP injection of OVA. The sensitized offspring were then exposed every day on days 14 to 21 to 30 min of 1% aerosol OVA.

After delivery, the offspring rats of each group were conventionally reared. From 6 to 8 weeks of age, according to the maternal origin, offspring rats (either sex) were randomly selected from every group and kept separate according to maternal treatment (*n* = 8 per group): offspring from control (OCP), asthmatic (OAP), NGF-treated (ONP) and anti-NGF-treated (OANP) pregnant rats. All groups of offspring were OVA-sensitized on days 0 and 7 with an IP injection of OVA, 200 mg of aluminum hydroxide, and 6 × 10^9^ heat-killed *Bordetella pertussis* in 1 mL of sterile saline. The sensitized offspring were then exposed every day on days 14 to 21 to 30 min of 1% aerosol OVA (Figure 1).

### Measurement of bronchial responsiveness in offspring

*In vivo* airway responsiveness to histamine was measured 24 h after the last OVA challenge using a whole-body plethysmograph (PLY 3211, Buxco Electronics, USA). Rats were treated for 2 min with increasing doses of histamine generated by an ultrasonic nebulizer, after which airway resistance was measured. Airway resistance was measured at baseline and at the peak of reaction. Histamine-induced bronchoconstriction was reported for the peak of the reaction as the percent of change in resistance from the baseline value [15,16].

### Tissue preparation and hematoxylin and eosin (H&E) staining

All offspring rats were anesthetized using an IP injection of 10% chloral hydrate (350 mg/kg). The left adrenal glands were excised and either snap-frozen at −80°C and fixed in 4% paraformaldehyde, or fixed in 2% glutaraldehyde, according to subsequent usage. Serum was obtained from each rat. After the pulmonary artery was perfused, the right middle lung lobe was collected from each rat, fixed in 4% paraformaldehyde and then embedded in paraffin. Lung and adrenal medulla tissue sections (4 μm) were stained with H&E, and the morphological changes were observed under a light microscope.

### Transmission electron microscopy in offspring

Adrenal medullae were fixed with 2% glutaraldehyde in 0.1 M cacodylate buffer, post-fixed in buffered 1% OsO_4_ for one hour, dehydrated in a graded ethanol series, and embedded in Epon-Araldite. Ultrathin sections (50 nm) were prepared from the different specimens and stained with uranyl acetate and lead citrate. Examinations of changes in ultrastructure were carried out with an H-7500 transmission electron microscope (TEM; Hitachi, Japan). Two pathologists who were blinded to the corresponding treatments assessed all samples.

### Immunohistochemistry

Tissues fixed in 4% paraformaldehyde at 4°C overnight were embedded in paraffin, sectioned, and mounted on slides. The tissue sections were then deparaffinized in toluene and rehydrated in increased concentrations of ethanol. Quenching of endogenous peroxidase activity, incubation with antibodies and peroxidase staining were performed according to the instructions from the manufacturer (ABC kit, Zhongshan Biological,Beijing, China). Tissue sections were exposed to anti-phenylethanolamine-n-methyl transferase (PNMT) antibody (Millipore USA, AB110, 1:2500) at 4°C overnight. Detection was achieved using a 3-amino-9-ethyl-carbazole (AEC kit, Zhongshan Biological,Beijing, China) to convert the chromogenic AEC substrate to a colored precipitate, and the nuclei were stained with Gill’s hematoxylin and observed under a light microscope (100×).

### Western blot analysis

Total proteins were extracted from tissue samples using RIPA lysis buffer (Takara, Japan). Thirty micrograms of protein were resolved via 10% sodium dodecyl sulfate-polyacrylamide gel electrophoresis (SDS-PAGE) and electrically transferred onto a polyvinylidene fluoride membrane at 120 V for 1.5 h. The transfer membrane was incubated with 0.05 g/mL skim milk at room temperature for 2 h. Rabbit anti-rat peripherin polyclonal antibody was then added (AB1530, 1:1000; Millipore) and incubated at 4°C overnight. After washing with PBS with Tween 20 (PBST), the membranes were incubated at 37°C for one hour with secondary antibodies (1:5000; Sigma) and visualized with an enhanced chemiluminescence system (Pierce Biotechnology, USA). β-actin was used as an internal control.

### Real-time PCR analysis

Total RNA was extracted from the adrenal tissues using Trizol (Invitrogen, Carlsbad, CA, USA). Reverse transcription of RNA was performed using a kit for first-strand complementary DNA synthesis (Promega, Madison, MI, USA). A PCR kit with SYBR Green PCR Master Mix (Takara, Shiga, Japan) was used to analyze the mRNA expression of peripherin and GAPDH. Real-time PCR was performed using the ABI PRISM 7900-HT Sequence Detection System (Applied Biosystems, Foster City, CA, USA). The primers were: peripherin, forward 5’-TGCTTCCCTAAGTTTAAAGACGAC-3' and reverse 5’-AGCTGTGAATAGAAGACTTGTCCA-3'; and GAPDH, forward 5’-TGACTTCAACAGCGACACCCA-3’ and reverse 5’-CACCCTGTTGCTGTAGCCAAA-3’. Real-time PCR data were analyzed according to the manufacturer’s instructions.

### Enzyme linked immunosorbent assay (ELISA)

Levels of EPI, corticosterone, and NGF in serum were quantified using the ELISA Kit. Commercially-available antibodies were applied according to the manufacturer’s recommendations (EPI, 0100–0009, Serotec, USA; corticosterone, 500651–96, Cayman, USA; NGF, BT555, BPB Biomedical, USA). The reactions were measured at 450 nm with an ELISA reader (Wellscan MK3, Labsystems, Finland).

### Statistical analysis

Data from offspring rats are presented as mean $\left(\overset{―}{x}\right)\pm \text{SD}$. One-way analysis of variance was used for multiple comparisons, followed by the Fisher’s protected (restricted) least significant difference test. A *P-*value less than 0.05 was considered statistically significant.

## Results

### Airway responsiveness to histamine in offspring

**Figure 2:**
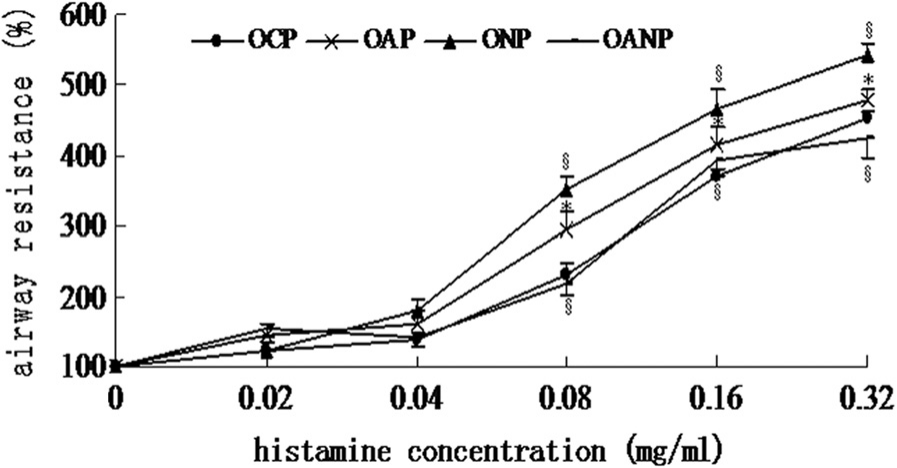
**Changes in airway resistance to histamine in offspring rats.** Rats were treated with increasing doses of aerosolized histamine. Afterwards, airway resistance was calculated as the percent of change in resistance at the peak of the reaction with reference to the baseline value. The values were expressed as mean ± SD (*n* = 8). ^*^Indicates a significant difference compared to the OCP group (*P* < 0.05). ^§^ Indicates a significant difference compared to the OAP group (*P* < 0.05).

At histamine concentrations ≥0.08 mg/mL, airway resistance significantly increased with increasing histamine concentrations in the OAP group, compared to the OCP group (all *P* < 0.05). Further increase in airway resistance was observed in the ONP group, while decreased airway resistance was found in the OANP group, both compared to that of the OAP group (all *P* < 0.05; Figure 2).

### Lung tissue morphology of offspring

**Figure 3:**
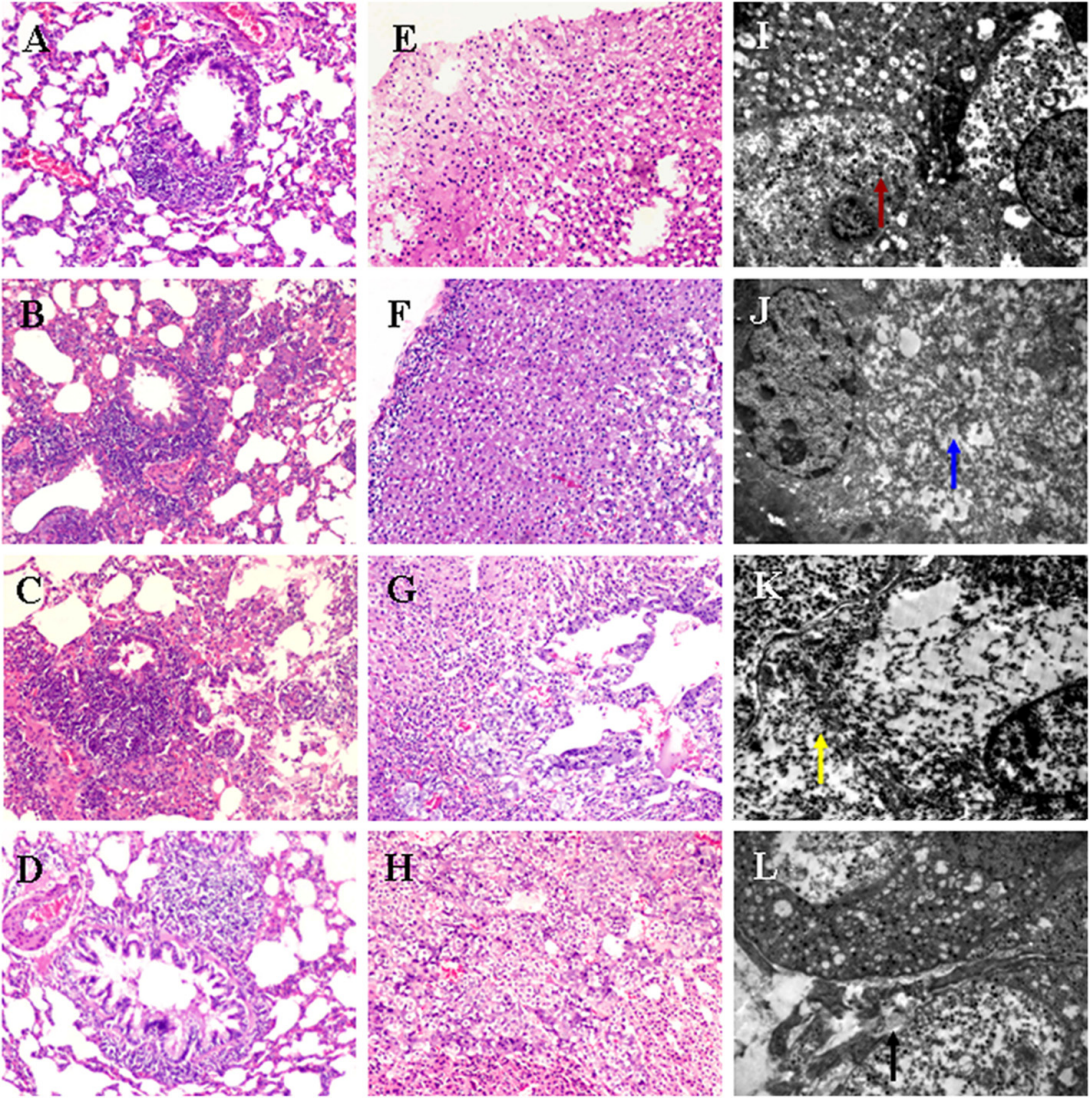
**Pathological changes in lung and adrenal medulla tissues in offspring rats.** Lung and adrenal medulla tissue sections (4 μm) from pups were stained with H&E, and the morphological changes were observed under a light microscope. Representative micrographs (100×) of lungs from OCP (**A**), OAP (**B**), ONP (**C**), and OANP rats (**D**). Representative micrographs (100×) of adrenal medulla in OCP (**E**), OAP (**F**), ONP (**G**), and OANP rats (**H**). Ultrathin sections (50 nm) from adrenal medullae were prepared from the different specimens and stained with uranyl acetate and lead citrate. Examinations of ultrastructural changes were carried out with an H-7500 TEM. Representative electron micrographs (10,000×) of adrenal medullae in OCP (**I**), OAP (**J**), ONP (**K**), and OANP rats (**L**). Colored arrows indicate chromaffin granules (red), swollen cytoplasm (blue), neurite-like processes (yellow), and deposition of collagen tissue (black).

Under the light microscope, infiltration of eosinophils (EOS) and neutrophils in the surrounding airway were found in each group. While serious pathological changes such as a mucous plug and swollen walls were observed in the OAP and ONP groups, these signs were relatively mild in the OCP and OANP group (Figure 3A-D).

### Adrenal medulla alteration in offspring

Under the light microscope, vacuolar degeneration and lipids were observed in the adrenal medullae of OCP rats, and these pathological changes were aggravated in the OAP and ONP groups. Compared with the OAP group, the extent of lesions was much less in the OANP group (Figure 3E-H).

With the electron microscope, lesions such as degranulation of chromaffin granules and swollen cytoplasm and mitochondria were observed in the OCP rats, but these were more severe in the OAP and ONP groups. In addition, neurite-like processes were observed in chromaffin cells of the ONP rats. Compared with the OAP group, Lesions were absent in the OANP group, and the collagen tissues was deposited in AMCC in the OANP rats (Figure 3I-L).

### Serum levels of EPI, corticosterone and NGF in offspring rats detected by ELISA

**Figure 4:**
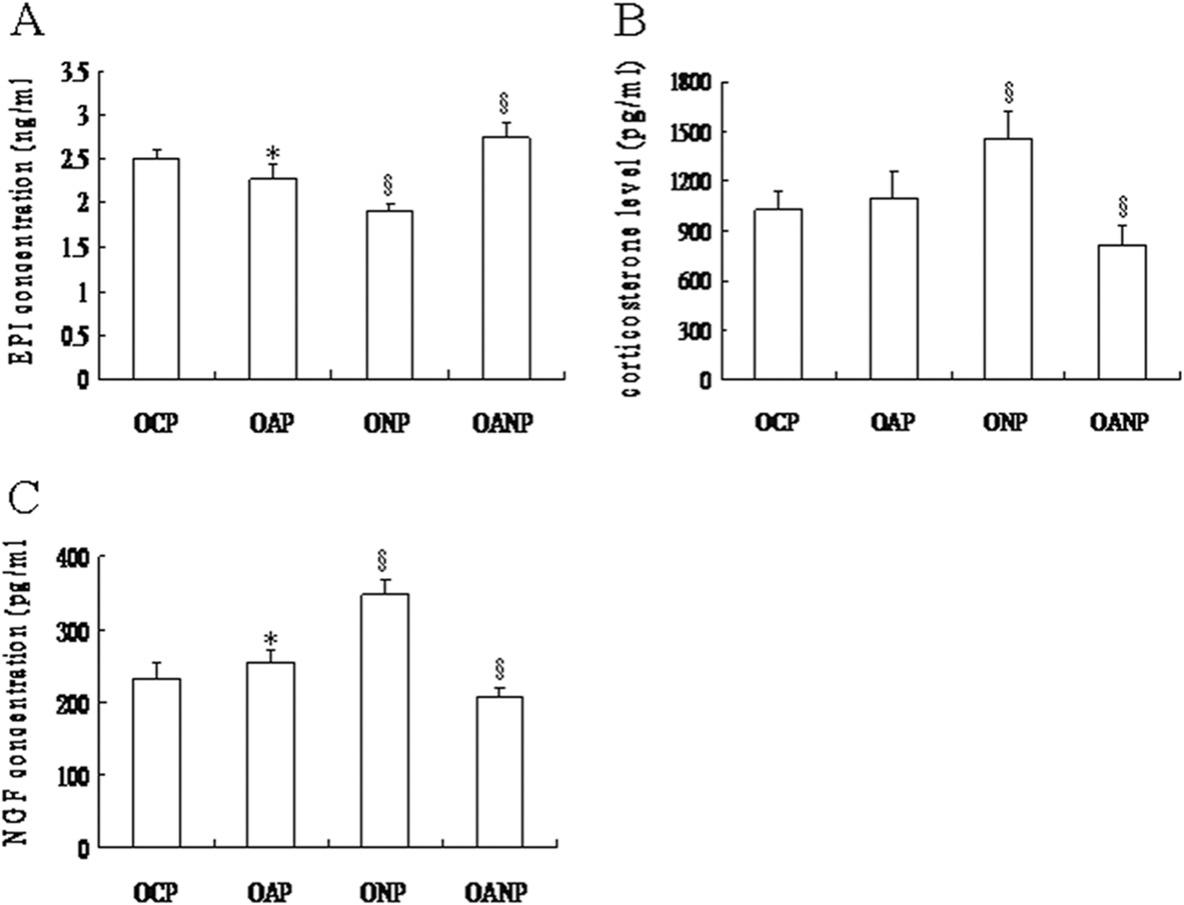
**Serum levels of EPI, corticosterone, and NGF in offspring rats.** Rat serum was collected and levels of EPI, corticosterone, and NGF were detected in each group with an ELISA kit. The values were expressed as mean ± SD (*n* = 8). ^*^Indicates significant difference compared to the OCP group (*P* < 0.05). ^§^ Indicates significant difference compared to the OAP group (*P* < 0.05).

Serum EPI levels of the OAP pups were significantly lower than those of the OCP group, while compared to the OAP pups, the EPI levels of the ONP rats were significantly lower still; the EPI levels of the OANP rats were significantly higher than those of the OAP pups (all *P* < 0.05; Figure 4A).

There was no significant difference found in serum corticosterone levels between the OCP and OAP groups (*P* > 0.05). However, compared with the OAP group the corticosterone level was significantly higher in the ONP rats, and significantly lower in the OANP rats (all *P* < 0.05; Figure 4B).

Similarly, serum NGF levels of OAP pups were significantly elevated compared to the OCP group, but those of the ONP (OANP) rats were significantly greater (less) than the OAP (all *P* < 0.05; Figure 4C).

### The expression of PNMT in adrenal medulla of offspring rats detected by immunohistochemistry

**Figure 5:**
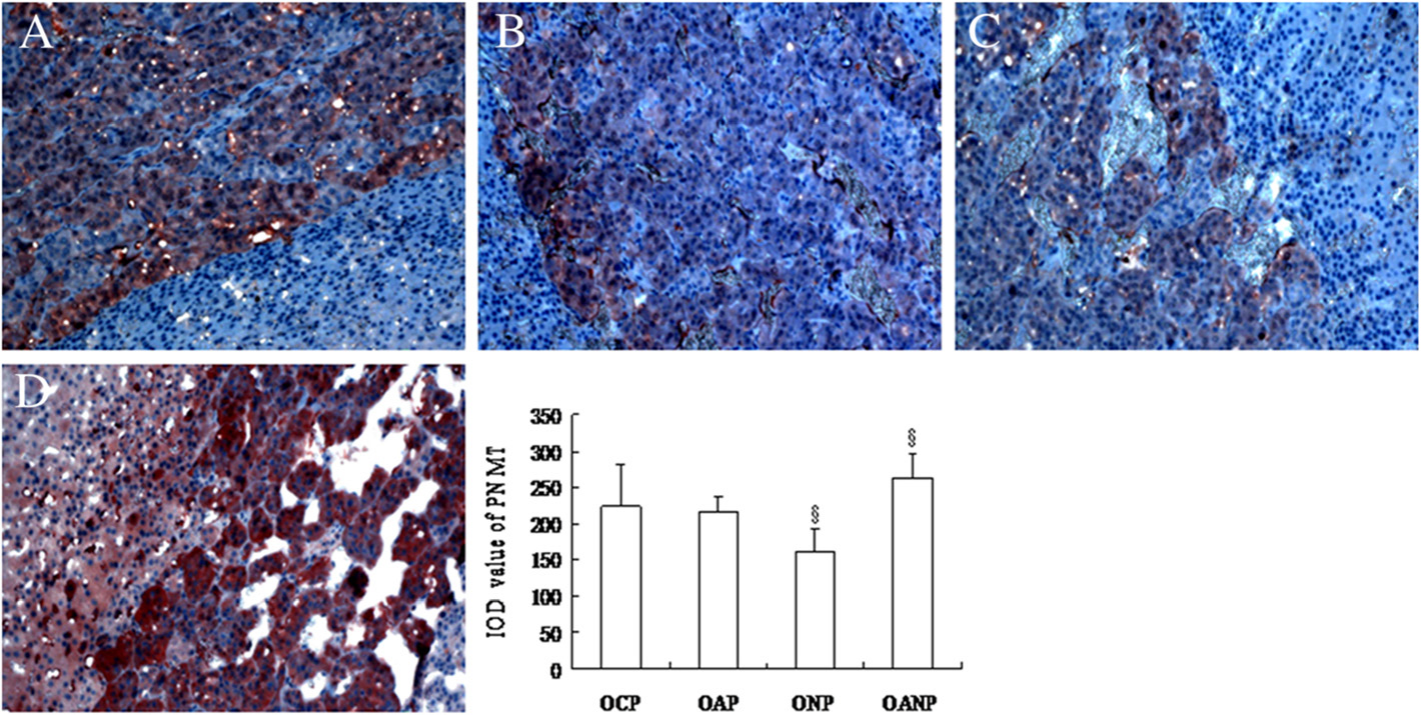
**Immunostaining of PNMT in adrenal medulla tissues in offspring rats.** Expression of PNMT in the adrenal medullae in each group was detected using immunohistochemistry. A representative sample of PNMT immunostaining (AEC, 100×) was presented in OCP (**A**), OAP (**B**), ONP (**C**), and OANP rats (**D**). The bar graph demonstrated the relative optical density results. The values were expressed as mean ± SD (*n* = 8). ^§^ Indicates significant difference compared to the OAP group (*P* < 0.05).

We found that immunostaining of PNMT mainly localized to the cytoplasm of adrenal medulla cells, and there was no significant difference in the expression of PNMT between the OCP and the OAP groups (*P* > 0.05). Compared with the OAP group, the expression of PNMT was decreased in the ONP rats, while increased in the OANP rats (*P* < 0.05; Figure 5).

### The expression of peripherin in adrenal medulla of offspring rats detected by real-time PCR and Western blot

**Figure 6:**
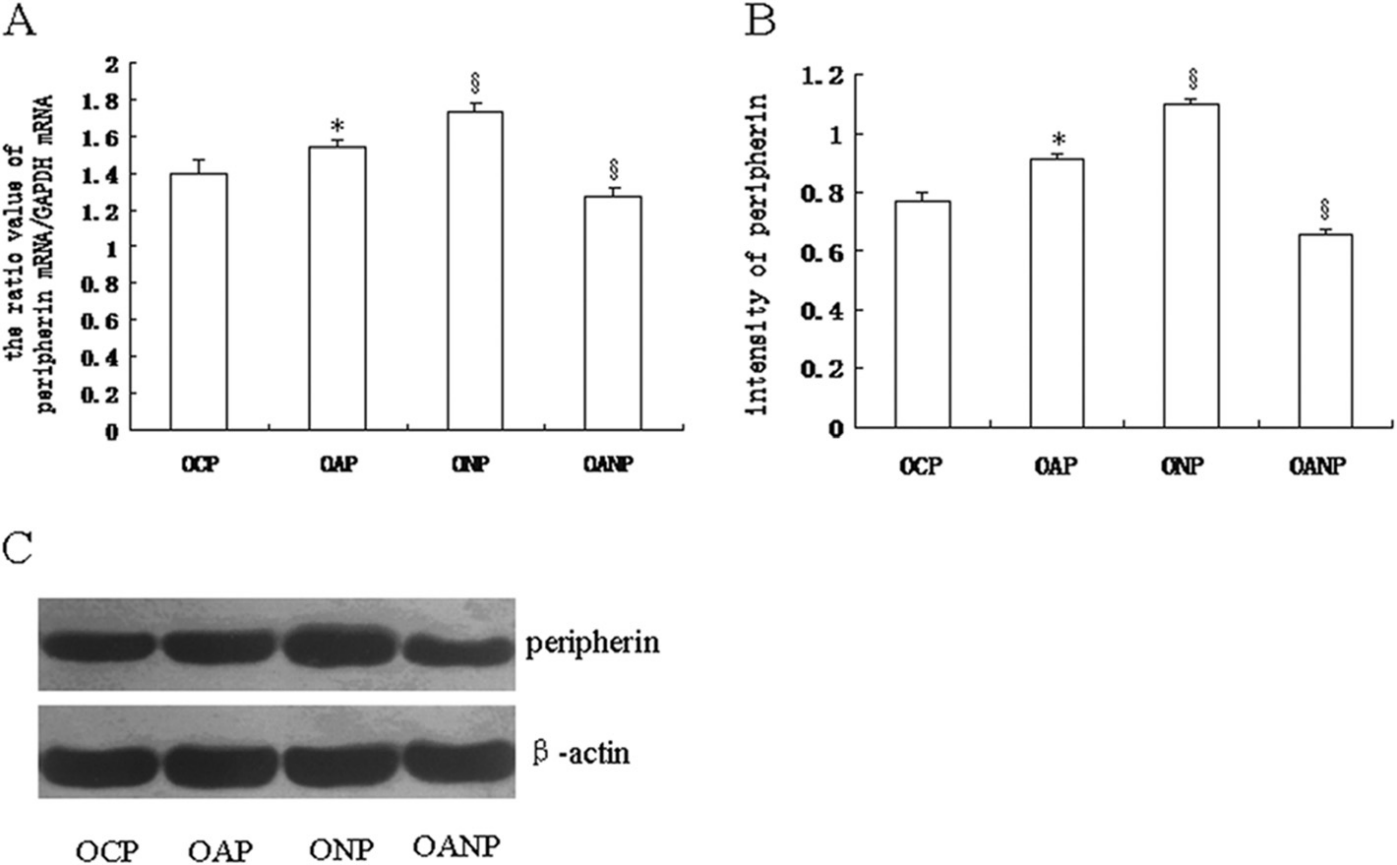
**Peripherin expression in adrenal medulla tissues in offspring rats.** Total RNA and protein were isolated from adrenal medulla, and the expressions of peripherin (mRNA and protein) in each group were detected by real-time RT-PCR (**A**) and Western blot (**B** and **C**), respectively. GAPDH or β-actin was used as internal controls for normalization purposes. The values were expressed as mean ± SD (*n* = 8). ^*^Indicates significant difference compared to the OCP group (*P* < 0.05). ^§^ Indicates significant difference compared to the OAP group (*P* < 0.05).

Compared with the OCP rats, a significant increase in peripherin mRNA and protein levels was found in OAP rats (all *P* < 0.05) as determined by real-time PCR and Western blot, respectively. A higher expression of peripherin mRNA and protein was noticed in the ONP rats compared to the OAP rats (all *P* < 0.05), while the expressions of both peripherin mRNA and protein in OANP rats were lower than in the OAP rats (all *P* < 0.05; Figure 6).

## Discussion

NGF, an important neurotrophic factor, was shown to increase in asthma and was related to the degree of bronchial hyperreactivity [11,17]. Hyperinnervation of the airway was observed in transgenic mice overexpressing NGF, and these mice were more sensitive to capsaicin-induced increases in airway resistance [18]. It has also been demonstrated that NGF increased sensory innervations, which led to changes in airway function [18]. Some studies have indicated that anti-NGF decreased airway hyperresponsiveness caused by allergen challenge, and this could be a therapeutic strategy for the treatment of allergic asthma [13,19-21]. We found recently that hyperinnervation of the adrenal medulla in asthmatic rats may contribute to the greater expression of NGF; the change of the adrenal medulla led to functional changes, which was associated with the transformation of AMCCs in asthma [15].

Because of a common origin from sympathoadrenal precursor cells, AMCCs can be transformed into sympathetic neurons under the stimulation of NGF, which is involved in neuronal growth, survival, and differentiation. An animal study indicated that prenatal injections of NGF, initiated in gestation and continued after birth with subcutaneous administration, produced massive transformation of chromaffin in sympathetic nerve cells of the adrenal medulla [22]. We recently found that asthma during pregnancy induced a tendency toward transformation from AMCCs to sympathetic neurons in offspring rats, which was also related to elevated NGF in the asthmatic dams [5]. These results suggested that the effect of NGF in asthma was strongly associated with the transformation of AMCCs.

In this study, all of pup groups (OCP, OAP, ONP, and OANP) were sensitized and challenged with OVA. In other words, all of pups were suffered from asthma, and the OCP pups were not the negative control pups but the asthmatic control pups. For this reason, the differences of NGF levels between OCP and OAP were existed (*P* < 0.05), but the changes may not be significant compared to that between the negative control and the asthmatic rats. It should be well noted that the differences in the function and ultrastructure of AMCCs were found among the four offspring groups from birth to adolescence prior to sensitization and challenge with OVA [5]. Compared with the pups form control pregnant rats, the pups from asthmatic pregnant rats showed a trend toward transformation of AMCCs [5]. When sensitized and challenged with OVA, the pathophysiology and phenotype changes of OAP rats were significant compared to that of OCP rats in this study. These results suggested that the susceptibility to OVA in asthmatic rats is also related to the phenotype state of AMCCs.

PNMT, the rate-limiting enzyme of EPI, is mainly expressed in adrenergic chromaffin cells but not in neurons. In this study, the expression of PNMT in OAP rats was lower than that of OCP rats, further reduction was found in ONP rats, and PNMT expression was enhanced in the OANP group. Interestingly, the level of PNMT in each group coincided with the expression of EPI. This indicated that the degree of injury in endocrine function of AMCCs was different among the four offspring groups, and related to the sensitivity to OVA.

Glucocorticoids are mainly secreted from the adrenal cortex. Similar to EPI, supplementing exogenous glucocorticoids is an effective treatment in asthma patients. Studies have shown that glucocorticoids could promote the expression of PNMT in the adrenal medulla, and that PNMT catalyzed the conversion of norepinephrine to EPI [23-25], which may be a mechanism underlying the value of glucocorticoids in the treatment of asthma. High concentration of glucocorticoids prevented fiber outgrowth from medullary chromaffin cells and inhibited the transformation of adrenal cells into neurons [26-28]. To a certain extent, the fate of AMCCs was determined by the balance of NGF and glucocorticoids in the milieu at any given time [28,29].

In the present study, although the glucocorticoid levels were increased in the OAP and ONP groups compared to the OCP, yet there was also a tendency toward transformation of AMCCs to sympathetic neurons in these groups. These results may suggest that NGF predominately played a role in this process. In other words, the volume of glucocorticoids was insufficient to counter that of the NGF, and the effect of glucocorticoids in alleviating bronchospasm may be obviated by elevated NGF.

During the development and regeneration of nerve cells, peripherin (a type III intermediate filament) plays an important role in establishing cellular architecture by regulating axon formation [30]. In fact, neurons with peripherin-siRNA demonstrated significantly impaired initiation, extension, and maintenance of neurites [30]. AMCCs were implicated in the active downregulation of the intermediate filament protein that occurs during embryonic development [8]. One study found that cells reactive to peripherin constituted a specific sub-population of AMCCs; this notion was supported by the fact that AMCCs, which highly expressed peripherin, had a distinct shape that was more like that of nerve cells [31].

In the present study, we showed that peripherin expression was higher in the OAP rats than in the OCP, and was higher still in the ONP, while reduced in the OANP. Accordingly, notable alterations in AMCCs such as neurite-like processes were found in the ONP rats. On the other hand, signs of reparation of AMCCs, such as collagen deposition, were found in the OANP rats. These results further indicate that the degree of alteration in AMCCs were significantly different among these offspring groups and suggests that peripherin plays a role in the transformation process.

## Conclusions

**Figure 7:**
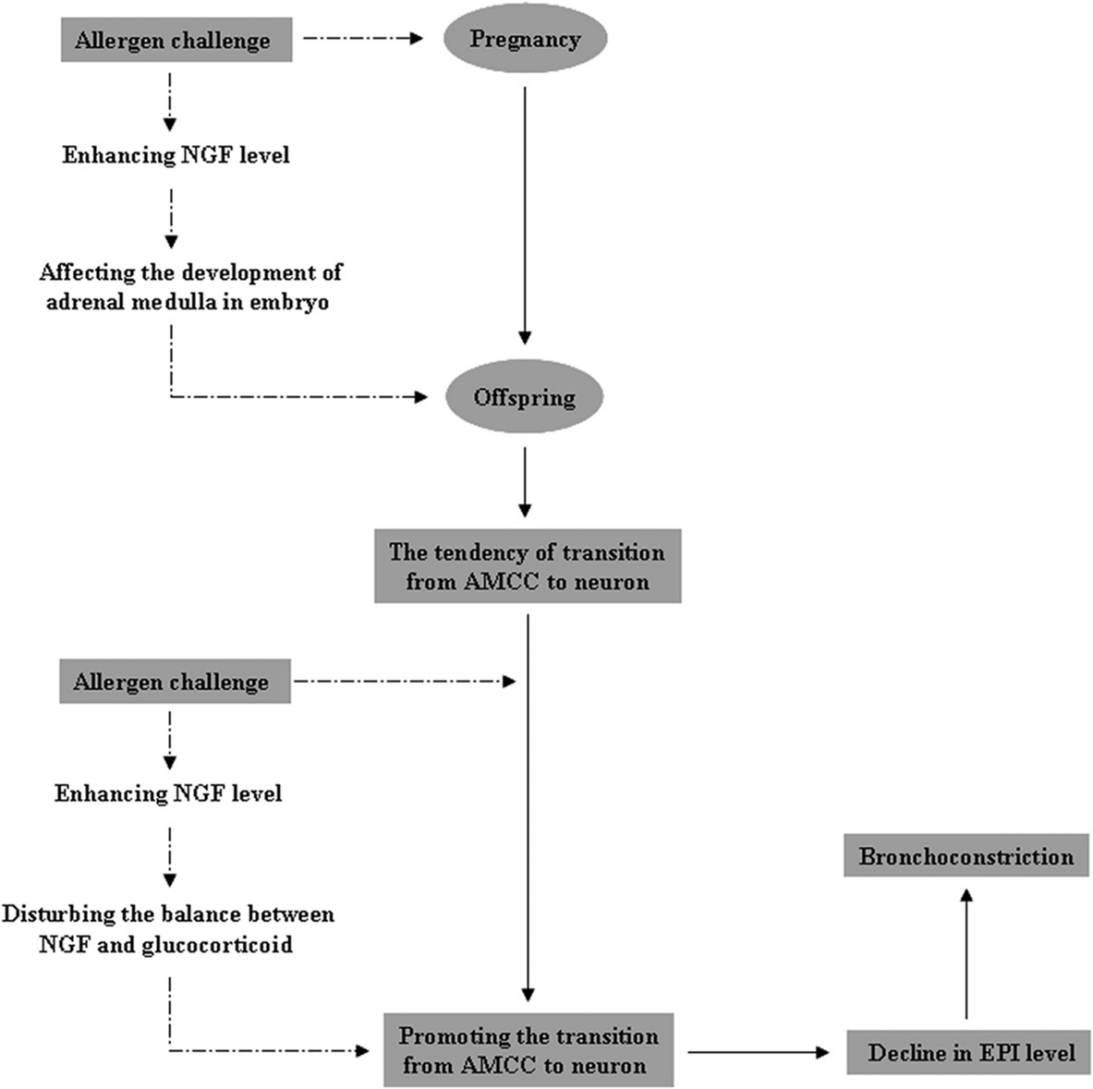
**Schematic summary of potential pathways for increasing asthma susceptibility in offspring rats from asthmatic dams.** Because AMCCs and sympathetic neurons share the same origin, the former retains the ability to transform from an endocrine to a neuronal phenotype. For this reason, a tendency for transformation from AMCCs to neurons appears in offspring from an asthmatic pregnancy under conditions of elevated NGF. When the offspring grow up and receive an allergen challenge, the enhancing NGF promotes the transformation of AMCCs. As a result, EPI secretion declines in the transformed AMCCs, which may lead directly to bronchoconstriction.

In summary, our results present evidence that asthma during pregnancy promotes asthma susceptibility in offspring, and the transformation from AMCC to neuron induced by NGF plays an important role in this process (Figure 7).

## Abbreviations

AMCC: Adrenal medulla chromaffin cell; EPI: Epinephrine; H&E: Hematoxylin and eosin; CP: Control pregnant rats; AP: Asthmatic pregnant rats; NP: NGF-treated pregnant rats; ANP: Anti-NGF-treated pregnant rats; OCP: Offspring from control pregnant rats; OAP: Offspring from asthmatic pregnant rats; ONP: Offspring from NGF-treated pregnant rats; OANP: Offspring from anti-NGF-treated pregnant rats; OVA: Ovalbumin; NGF: Nerve growth factor; PNMT: Phenylethanolamine-n-methyl transferase.

## Competing interests

There are no competing interests involved in the article.

## Authors’ contributions

JTF and CPH participated in the conception and design of the study. XMW, XZL, YQZ and LQ performed the animal study, ELISA, lung histology, immunochemistry, RT-PCR and western blotting. JTF and CPH analyzed the data and drafted the manuscript. All authors have read and approved the final manuscript.
